# The Genomic Basis of Postponed Senescence in *Drosophila melanogaster*

**DOI:** 10.1371/journal.pone.0138569

**Published:** 2015-09-17

**Authors:** Megan Ulmer Carnes, Terry Campbell, Wen Huang, Daniel G. Butler, Mary Anna Carbone, Laura H. Duncan, Sasha V. Harbajan, Edward M. King, Kara R. Peterson, Alexander Weitzel, Shanshan Zhou, Trudy F. C. Mackay

**Affiliations:** 1 Department of Biological Sciences, North Carolina State University, Raleigh, North Carolina, 27695, United States of America; 2 Program in Genetics, North Carolina State University, Raleigh, North Carolina, 27695, United States of America; 3 W. M. Keck Center for Behavioral Biology, North Carolina State University, Raleigh, North Carolina, 27695, United States of America; Lancaster University, UNITED KINGDOM

## Abstract

Natural populations harbor considerable genetic variation for lifespan. While evolutionary theory provides general explanations for the existence of this variation, our knowledge of the genes harboring naturally occurring polymorphisms affecting lifespan is limited. Here, we assessed the genetic divergence between five *Drosophila melanogaster* lines selected for postponed senescence for over 170 generations (O lines) and five lines from the same base population maintained at a two week generation interval for over 850 generations (B lines). On average, O lines live 70% longer than B lines, are more productive at all ages, and have delayed senescence for other traits than reproduction. We performed population sequencing of pools of individuals from all B and O lines and identified 6,394 genetically divergent variants in or near 1,928 genes at a false discovery rate of 0.068. A 2.6 Mb region at the tip of the *X* chromosome contained many variants fixed for alternative alleles in the two populations, suggestive of a hard selective sweep. We also assessed genome wide gene expression of O and B lines at one and five weeks of age using RNA sequencing and identified genes with significant (false discovery rate < 0.05) effects on gene expression with age, population and the age by population interaction, separately for each sex. We identified transcripts that exhibited the transcriptional signature of postponed senescence and integrated the gene expression and genetic divergence data to identify 98 (175) top candidate genes in females (males) affecting postponed senescence and increased lifespan. While several of these genes have been previously associated with *Drosophila* lifespan, most are novel and constitute a rich resource for future functional validation.

## Introduction

Lifespan and senescence (the post-reproductive decline in survival and fertility with advancing age) vary enormously within and among taxa, with some organisms attaining exceptional longevity and negligible senescence [1–3]. The question of why lifespan is limited and why there is variation in aging within species is addressed in general terms by evolutionary theory. Natural selection declines with age [4], so mutations with late age-specific deleterious effects are nearly neutral with respect to natural selection and can accumulate in populations at appreciable frequencies [5]. In addition, mutations with beneficial effects early in life but detrimental effects later in life will accumulate [6,7]. Both classes of mutations, in addition to unconditionally deleterious mutations affecting survival and fertility at all ages, will result in segregating genetic variation for lifespan in natural populations.

Indeed, estimates of the heritability of lifespan range from 10–30% in humans and other organisms [1,2,8–16], and quantitative trait loci (QTLs) affecting lifespan have been mapped in *C*. *elegans* [17–19], *Drosophila* [20–31], mice [32–34] and humans [35–39]. However, with a few exceptions [40–42], naturally segregating loci affecting lifespan have not been mapped to the level of individual genes. In contrast, studies assessing changes in gene expression with age, transgenic manipulation of candidate genes and direct screens for mutations affecting lifespan, largely in model organisms, have identified multiple genetic mechanisms affecting lifespan, some of which are ‘public’ [3,43] and common to multiple taxa. One such general mechanism of aging is the regulation of metabolism, in particular components of the insulin or insulin-like signaling pathway [44–59]. The increase in lifespan associated with decreased insulin signaling may be mechanistically related to the increase in life span conferred by dietary restriction [60–64]. Other general mechanisms of aging inferred from analyses of model organisms include genes affecting the ability to detoxify reactive oxygen species [65–69], reproduction [21,48,70–77], gene silencing [78–80], telomere integrity [81], DNA repair and replication [82–84], mitochondrial [85,86] and membrane function [87], resistance to heat, starvation and other environmental stressors [88–94], sensory perception [95,96], and immune response [97–100]. Despite these insights, we do not know to what extent these genes harbor polymorphisms affecting naturally segregating variation in lifespan within populations.

A powerful alternative to mapping QTLs by linkage and association analysis is to track changes in allele frequency between replicated populations undergoing laboratory evolution via natural or artificial selection [101–103]. This approach has the potential to identify causal genes and even variants affecting the trait of interest that are in common between all evolved populations under favorable experimental conditions (sufficient replication, large and genetically diverse populations, many generations of evolution, and genome wide variant detection by whole genome sequencing) [104–107]. Combining genetic divergence analyses with changes in gene expression can further aid in identifying causal genes and provide biological context to genome wide polygenic divergence [104,105]. *D*. *melanogaster* populations respond rapidly to laboratory selection for increased lifespan [15,104,108–110], facilitating the application of the ‘evolve and re-sequence’ approach to this trait. Here, we combine pooled DNA sequencing with RNA sequencing at two chronological ages to quantify the genetic and genomic responses of five replicate lines selected for postponed senescence via later reproduction (Old, or O lines) relative to five unselected control lines (Base, or B lines) [109]. We identify many novel candidate genes affecting increased lifespan and postponed senescence, many of which are orthologous to human genes.

## Materials and Methods

### *Drosophila* stocks

Ten *D*. *melanogaster* lines generated by Rose [109] were used in this study. Five lines were selected for delayed reproductive senescence (O_1_, O_2_, O_3_, O_4_, O_5_) and five were unselected (B_1_, B_2_, B_3_, B_4_, B_5_). The B and O line stocks were maintained in 14-day and 70-day generations, respectively, as described previously [111]. All lines were maintained at 25°C on cornmeal-molasses-agar medium (cornmeal, 65 g/L; molasses, 45 ml/L; yeast, 13 g/L) under a 12:12 hour light:dark cycle. These flies were a generous gift of Dr. Philip Service.

### Life history phenotypes and statistical analyses

#### Lifespan

To minimize larval density effects, experimental flies were produced for each line by allowing 6 males and 6 females to mate and lay eggs for one day in vials containing 10 ml culture medium. Offspring from these vials were collected at 1–3 days post-eclosion for lifespan assays. Lifespan was assessed for each population using 50 replicate vials, each containing 3 males and 3 females and 5 ml culture medium. Flies were transferred without anesthesia to new vials containing 5 ml of fresh food every 2–3 days; dead flies were removed from the vials upon observation. Deaths were recorded every 1–3 days until all individuals were deceased. We performed two-way factorial mixed-model analyses of variance (ANOVA) of lifespan separately for males and females of form: *Y* = *μ* + *P* + *L*(*P*) + *Rep*(*P*×*L*) + *ε*, where *P* is the fixed effect of population (B vs. O), *L* and *Rep* are the random effects of line and replicate, respectively, and *ε* is the residual (error) variance. Parentheses indicate nested effects. ANOVAs were performed using SAS software version 9.4 [112].

#### Phototaxis

Parental flies were reared as described above for lifespan. Offspring used in phototaxis assays were maintained in bottles with 50 males and 50 females per bottle and transferred to fresh bottles every 2–3 days. Phototaxis was assessed in the countercurrent apparatus [113] for three replicates of 50 flies per sex per line at one, two, three and four weeks of age. Flies were allowed to recover overnight from CO_2_ exposure, and dark-adapted for 30 minutes prior to performing the assay in a dark room between 9:00–11:30 am. To assess phototaxis, flies were tapped to the bottom of the first start tube and the apparatus was laid horizontally with the distal tubes 5 cm away from a 15 W fluorescent light. The flies were given 15 seconds to reach the distal tube, and the procedure was repeated 7 more times per trial. Thus, each fly received a score between 1 (did not move toward the light in the first tube) and 8 (moved towards the light 7 times). We performed two-way factorial mixed-model ANOVAs of phototaxis separately for males and females of form: *Y* = *μ* + *P* + *A* + *P*×*A* + *L*(*P*) + *A*×*L*(*P*) + *Rep*(*A*×*P*×*L*) + *ε*, where *P* and *A* are the fixed effects of population and age, *L* and *Rep* are the random effects of line and replicate, respectively, and *ε* is the residual (error) variance. Parentheses indicate nested effects.

#### Capillary feeding (CAFÉ) assay

Parental and experimental flies were reared as described above for lifespan. Food consumption was assessed using a modified version of the CAFÉ assay [114] at one, two, three and four weeks of age. Eight flies of the same sex, line and age were anesthetized with CO_2_ and placed into each of six replicate vials containing 2 ml of 1.5% non-nutritive agarose and three 5 μL capillary tubes (Kimble Glass Inc.) containing a 4% (weight/volume) sucrose solution inserted through a foam plug. The capillary tubes were capped with mineral oil to minimize evaporation. The vials were placed in a transparent plastic container in which high humidity is maintained with open containers of water at 25°C and the flies were allowed to feed on the sucrose solution for 24 hours. After the first 24 hours of acclimation, the capillaries were removed and three fresh capillaries with sucrose solution were added to each vial. The flies were then given another 24 hours to feed on the sucrose solution after which the capillaries were marked to indicate the amount of food consumed and removed and the number of surviving flies was recorded. The amount of sucrose solution consumed was measured in millimeters to the closest 0.5 mm and adjusted to μl per fly per vial after correction for evaporation using control vials containing no flies in the same humidity chamber. We performed two-way factorial mixed-model ANOVAs of food consumption separately for males and females of form: *Y* = *μ* + *P* + *A* + *P*×*A* + *L*(*P*) + *A*×*L*(*P*) + *ε*, where *P*, *A*, *L* and *ε* are as defined above for phototaxis. Parentheses indicate nested effects.

#### Chill coma recovery time

Parental and experimental flies were reared as described above for phototaxis. Chill coma recovery time [115] was assessed at one, two, three and four weeks of age with 50 flies per sex, line and age. We anesthetized flies using CO_2_ and let them recover for 24 hours prior to the assays. We then quantified chill coma recovery by transferring (without anesthesia) flies to empty vials, and placing them on ice for three hours. We transferred the flies to room temperature, and recorded the time it took for each individual to right itself and stand on its legs. We performed two-way mixed model ANOVAs as described for food consumption.

#### Productivity

Parental and experimental flies were reared as described for phototaxis. Productivity was assessed at one, two, three and four weeks of age with 10 vials containing 10 ml culture medium and 3 males and 3 females per line and age. Experimental animals were anesthetized using CO_2_ and given 24 hours to recover prior to setting up the experimental vials. Flies were allowed to lay eggs for 24 hours, and the total number of emerging progeny was counted every day for a total of 16 days. We performed two-way mixed model ANOVAs as described for food consumption.

### B and O sequence divergence

Genomic DNA was extracted from 100 females of each line using Genomic-Tip 100/G columns (Qiagen Inc.), following homogenization using liquid nitrogen with a mortar and pestle. Genomic DNA was sequenced on the Illumina GAIIx platform by 68bp paired-end sequencing at the Genome Sciences Laboratory, North Carolina State University. Sequence reads were aligned to the BDGP5 reference genome using BWA (version 0.6.2) [116]. Alignments were locally realigned, marked for PCR duplicates, and base qualities were recalibrated using GATK (version 2.4) [117] and Picard Tools (version 1.89) [117]. Subsequently, uniquely mapped reads were piled up at each genomic position to identify putative SNPs. A polymorphic site was considered for further analysis if it passed the following filters: (1) at least 10X coverage by bases with Phred scale quality > 13; (2) bases passing the quality filter constituted at least 80% of all bases at the site; (3) no more than 250X coverage; (4) maximally 5% reads at the site represented indels; (5) the two most frequent alleles accounted for more than 95% of all alleles; (6) the minor allele frequency was at least 5% in at least one of the ten samples; (7) the Chernoff bound of the *P*-value for testing polymorphism versus sequencing error was smaller than 10^−5^; and (8) the Fisher’s exact test for strand bias had *P* > 10^−5^. Divergence of allele frequency between O and B was tested using *t*-tests at each site individually, requiring that at least three O lines and three B lines had estimable allele frequencies.

### Gene expression analysis

#### RNA extraction and sequencing

Flies were reared exactly as described for lifespan. We collected 80 samples for RNA sequencing, all between 1–3 pm. There were two replicate samples of 50 3–5 day old males and females (week 1 samples) and 50 33–35 day old males and females (week 5 samples) from each line, with one exception. The B_1_ females are shorter-lived than the other B lines; therefore the old B_1_ females were 26–28 days old (4 weeks) in order to obtain sufficient flies. Flies were flash frozen over dry ice and stored at -80°C. Total RNA was extracted with Trizol (Life Technologies, California, USA) and an RNAeasy Mini Kit (Qiagen, Limburg, Germany). Ribosomal RNA (rRNA) was removed using a Ribo-Zero™ Gold Kit (Epicentre, Wisconsin, USA) with 5 ug total RNA input. Depleted mRNA was fragmented and converted to first strand cDNA. During the synthesis of second strand cDNA, dUTP instead of dTTP was incorporated to label the second strand cDNA. cDNA from each RNA sample was used to produce barcoded cDNA library using NEXTflex™ DNA Barcodes (Bioo Scientific, Texas, USA) with an Illumina TrueSeq compatible protocol. Library size was selected using Agencourt Ampure XP Beads (Beckman Coulter, Indiana, USA) and centered around 250 bp with an approximate insert size of 130 bp. Second strand DNA was digested with Uracil-DNA Glycosylase before amplification to produce directional cDNA libraries. Libraries were quantified using Qubit dsDNA HS kits (Life Technologies) and a 2100 Bioanalyzer (Agilent Technologies, California, USA) to calculate molarity. They were then diluted to an equal molarity, re-quantified, and 16 libraries were pooled. Pooled library samples were quantified again to calculate the final molarity, denatured, and diluted to 14 pM. Pooled library samples were clustered on an Illumina cBot and sequenced on an Illumina Hiseq2500 using 125 bp single-read v4 chemistry.

#### Transcriptome assembly and analysis

RNA sequences were demultiplexed using the Illumina bcl2fastq program (version 1.8.4) and summarized as follows: there was an average of 17,447,402 reads generated per sample (min. 14,194,039 reads/sample, max. 24,652,714 reads/sample), and 93% of reads had a base call accuracy of ≥ 99.9%. Read quality was assessed using the FastQC program (version 0.11.2) (http://www.bioinformatics.babraham.ac.uk/projects/fastqc/). The reads were pre-processed using Cutadapt (version 1.6) [118] to remove residual adapter sequences. Ribosomal RNA (rRNA) sequences were removed by aligning the sequencing reads using TopHat (version 2.0.13) [119] to known rRNA sequences downloaded from GenBank [120] and FlyBase (Dmel Release 5.57) [121]. The remaining reads were processed following the Tuxedo suite pipeline [122]. The sequencing reads were aligned to the *D*. *melanogaster* reference transcriptome and reference genome (FlyBase Dmel Release 5.57) using default parameters, allowing for up to 5 mismatches per read. Alignment rates were highly variable with an average alignment rate of 86%. We ruled out sample degradation, human contamination, and sequencing lane errors as the cause for the observed mapping rate variability, and inferred that natural sources of bacteria and other environmental components were the most likely explanation. After alignment, reads were assembled against the FlyBase reference transcriptome for gene-level analysis of annotated genes using Cuffquant (version 2.2.1) and default parameters, allowing for an increased number of fragments per locus (-max-bundle-frags increased to 10,000,000). The resulting expression values were geometric mean normalized using Cuffnorm (version 2.2.1) [123]. For the analysis of unannotated transcripts, transcriptome assembly was first performed using Cufflinks (version 2.2.1) without providing the annotated Flybase transcriptome; all other parameters were maintained. The resulting transcriptomes, from all 80 samples, were merged using Cuffmerge (version 2.2.1) to generate a high-confidence, sample-specific, reference transcriptome. Sample transcriptomes were then re-assembled against the generated reference transcriptome and expression levels were quantified using Cuffquant. The expression values were then geometric mean normalized using Cuffnorm. Finally, unannotated genes were identified for analysis by selecting expressed loci classified as ‘unknown, intergenic’ (transfrag class code u). After normalization, any gene (annotated or unannotated) whose mean expression value was 0 or whose maximum expression value was < 1 was dropped from the analysis.

We performed a factorial mixed effect ANOVA of the normalized gene-level count data using the following model: *Y* = *μ* + *A* + *S* + *P* + *A*×*S* + *A*×*P* + *S*×*P* + *A*×*S*×*P* + *L*(*P*) + *ε*, where *S* denotes the fixed effect of sex and all other terms are as defined above. We used the Benjamini and Hochberg method [124] of controlling the false discovery rate (FDR), and considered an FDR of < 0.05 for any term in the ANOVA to be significant. Over 94% of genes had a significant sex main effect or interaction term. Therefore, we performed ANOVAs separately for males and females for each gene expression trait using the model: *Y* = *μ* + *A* + *P* + *A*×*P* + *L*(*P*) + *ε*. All statistical analyses were conducted using SAS software [112]. Gene annotation and identification of human orthologs was done using Ensembl databases [125].

### Gene ontology (GO) and functional annotation analyses

We performed gene ontology and functional annotation analyses for genetically divergent genes and genes significant for the fixed effects in the ANOVAs of gene expression using the functional annotation cluster tool in DAVID (version 6.7) [126,127]. If a gene subset of interest was larger than 3,000 (a DAVID-set threshold), then the top 3,000 most significant genes were used in the analysis. An enrichment score of 5 (corresponding to a geometric mean normalized *P*-value threshold of 1E-5) was used as the significance cutoff.

## Results

The O and B lines were derived from an outbred laboratory population founded by wild-caught animals from South Amherst, MA, USA in 1970, and maintained at large population size for 130 generations prior to establishing the experimental populations [109]. Thus, the base population had been adapted to laboratory conditions and should have reached global quasi-linkage equilibrium before the establishment of the B and O populations in February 1980 [109]. At the time the experiments described here were conducted, the B populations had been continually maintained at 14 day discrete generation intervals for over 850 generations and the O populations at 70 day generation intervals for over 170 generations, since 1996 in the Mackay laboratory. Population sizes each generation are of the order of one to two thousand individuals for all lines. Thus, these lines have many desirable properties for analysis by the ‘evolve and re-sequence’ approach. The populations are large and genetically heterogeneous, well-replicated, and have undergone many generations of evolution under controlled environmental conditions.

### Phenotypic characterization of O and B lines

**Fig 1:**
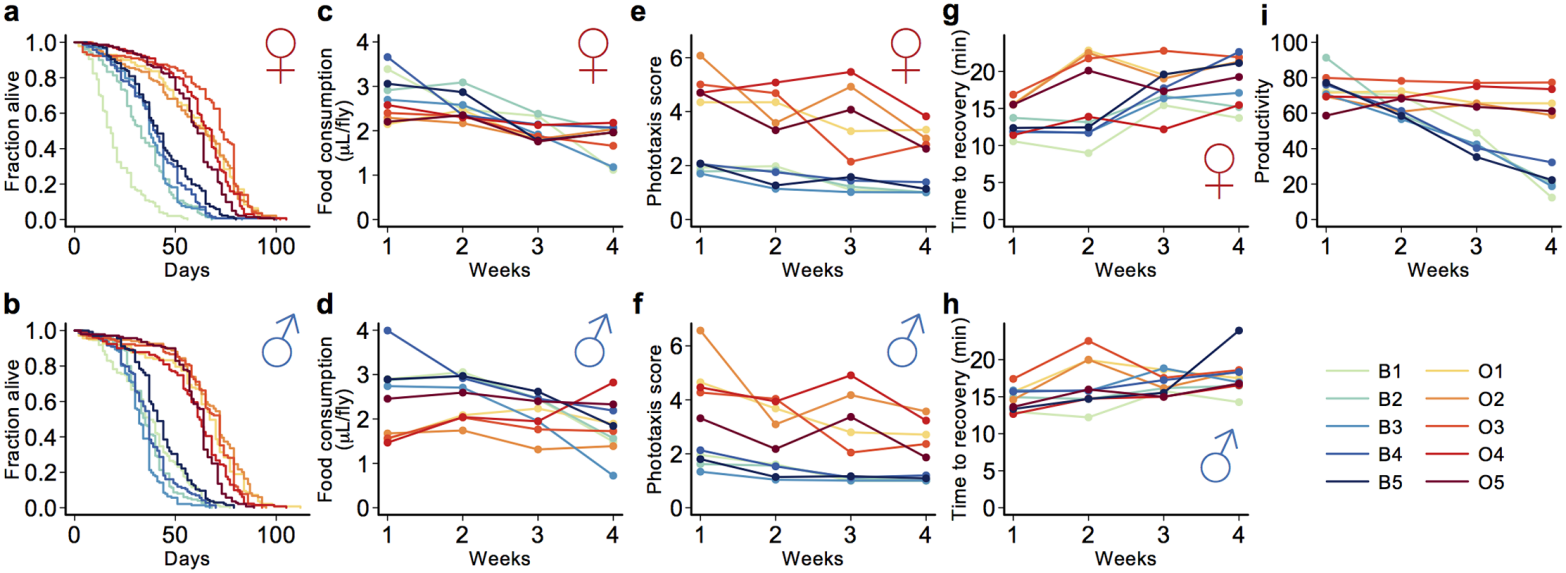
Phenotypic differentiation and senescence in the O and B populations. (**A**) Female lifespan. (**B**) Male Lifespan. (**C**) Female food consumption. (**D**) Male food consumption. (**E**) Female phototaxis. (**F**) Male phototaxis. (**G**) Female chill coma recovery time. (**H**) Male chill coma recovery time. (**I**) Productivity. The B and O lines are color coded as indicated.

The O and B populations are highly divergent for lifespan (Fig 1A and 1B; S1 Table). On average, O lines live 70% longer than B lines (62.1 days vs. 35.7 days, respectively). Since the O lines were selected for postponed reproductive senescence, we assessed productivity and other fitness-related traits of all lines at one, two, three and four weeks of age (Fig 1C–1I, S1 Table). The productivity of O lines is higher than that of B lines averaged over all ages, and the B lines exhibit marked reproductive senescence while the O lines do not (Fig 1I). Averaged over all ages, the B lines consume more sucrose in the CAFÉ assay than the O lines, and food consumption generally declines with age. However, this decline is greatest for the B lines (Fig 1C and 1D). The O lines respond robustly in the phototaxis assay and this tendency declines with age, but surprisingly, the B lines barely move towards light, even at week one (Fig 1E and 1F). With respect to the CAFÉ and phototoaxis assays, B lines have a ‘couch potato’ phenotype, eating a lot and moving little, while the O lines eat less and are more motile (at least in response to a light stimulus). The time to recover from a chill induced coma increases with age, but there is little differentiation between the O and B lines averaged over all ages. However, the B lines display more senescence for this trait than do the O lines, particularly in females (Fig 1G and 1F). Thus, response to continuous selection for postponed senescence has persisted, and is accompanied by correlated responses in lifespan and other fitness traits, and delayed senescence for traits other than reproduction.

### B and O sequence divergence

**Fig 2:**
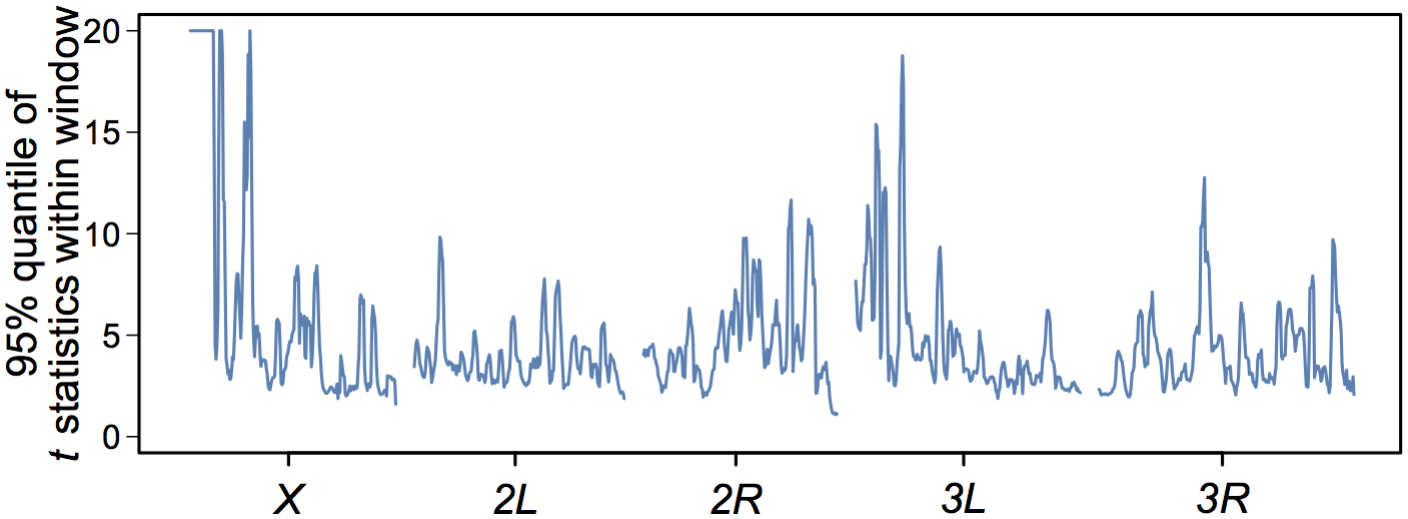
Genome-wide DNA divergence between O and B lines. The 95% quantiles of *t*-statistics within sliding windows of size 0.5Mb (by a sliding size of 0.1Mb) are plotted along the chromosome arms. The *t*-statistics are capped at 20 to enhance visualization of smaller differences.

We performed population sequencing of pools of individuals from all B and O lines. We identified 432,580 single nucleotide polymorphisms (SNPs) segregating in these lines, and performed simple *t*-tests to assess differences in allele frequency between the populations (Fig 2, S2 Table). At a lenient reporting *P*-value threshold of *P* ≤ 10^−3^, we identified 6,394 variants in or near 1,928 genes (for a false discovery rate of FDR = 0.068). A total of 450 variants in or near 413 genes exceeded a rigorous Bonferroni correction for multiple tests. The majority of the highly significant variants were located in a 2.6 Mb region at the tip of the *X* chromosome, which contained many variants fixed for alternative alleles in the two populations, suggestive of a hard selective sweep and long range linkage disequilibrium (LD) [128]. Several of the genes with differences in SNP allele frequencies between the B and O populations had previously been implicated to affect lifespan (e.g., *Cat* [65], *Cct1* [87], *cpo* [129], *Dhc64c* [130], *Eip75B* [131], but the vast majority are novel candidates. We performed Gene Ontology (GO) enrichment analyses [126,127], excluding genes in the 2.6 Mb region of the X chromosome because the high degree of LD in this region precludes identifying candidate genes. The genetically divergent genes are highly enriched for biological process categories involved in development and differentiation, in particular the development and function of the nervous system (S3 Table).

### Gene expression analysis

We assessed genome wide gene expression of O and B lines at one and five weeks of age, separately for males and females, using RNA sequencing (S4 Table). Week one corresponds to 11% and 20% of the average lifespan of O and B lines, respectively; while week five corresponds to 56% of the average lifespan of O lines and 98% of the average lifespan of B lines. We performed a three-way factorial analysis of variance (ANOVA) on each gene expression trait to partition the variation in gene expression between the main effects of sex, age, population and all interactions. We used a false discovery rate of FDR < 0.05 to account for multiple tests. We found that sex was significant for 94% of genes as a main effect or interaction term, consistent with previous analyses documenting substantial sexual dimorphism of gene expression [23,130,132,133]. Therefore we performed two-way ANOVAs to partition variance of gene expression between the main effects of age, population and the age by population interaction, as well as among lines within each population, for all annotated genes with detectable expression (15,586 in females and 16,174 in males). A summary of the number of significant genes for each term in the ANOVAs is provided in Table 1 and full ANOVA results are given in S5 and S6 Tables.

**Table 1:** Numbers of significant (FDR < 0.05) annotated genes for each term in the ANOVA models of gene expression.

Term	Female	Male	Both sexes
Age	4,145 (27%)	9,853 (61%)	3,085 (20%)
Age up-regulated (W1 < W5)	1,999	4,231	1,300*
Age down-regulated (W1 > W5)	2,146	5,622	1,360*
Population	780 (5%)	1,011 (6%)	178 (1%)
Population B < O	568	438	115*
Population B > O	212	573	58*
Age × Population	3,171 (20%)	4,998 (31%)	1,276 (8%)
Total number of tested genes	15,586	16,174	15,563

W1: Week 1; W5: Week 5.

* These numbers do not sum to the total numbers in both sexes because of differences in the directionality of effects across sexes.

3,085 genes have a significant age effect in both the male and female dataset but only 2660 have an effect in the same direction. The remaining 425 genes are up-regulated in one sex and down-regulated in the other. Likewise, 5 genes have opposite effects in the B and O populations.

**Fig 3:**
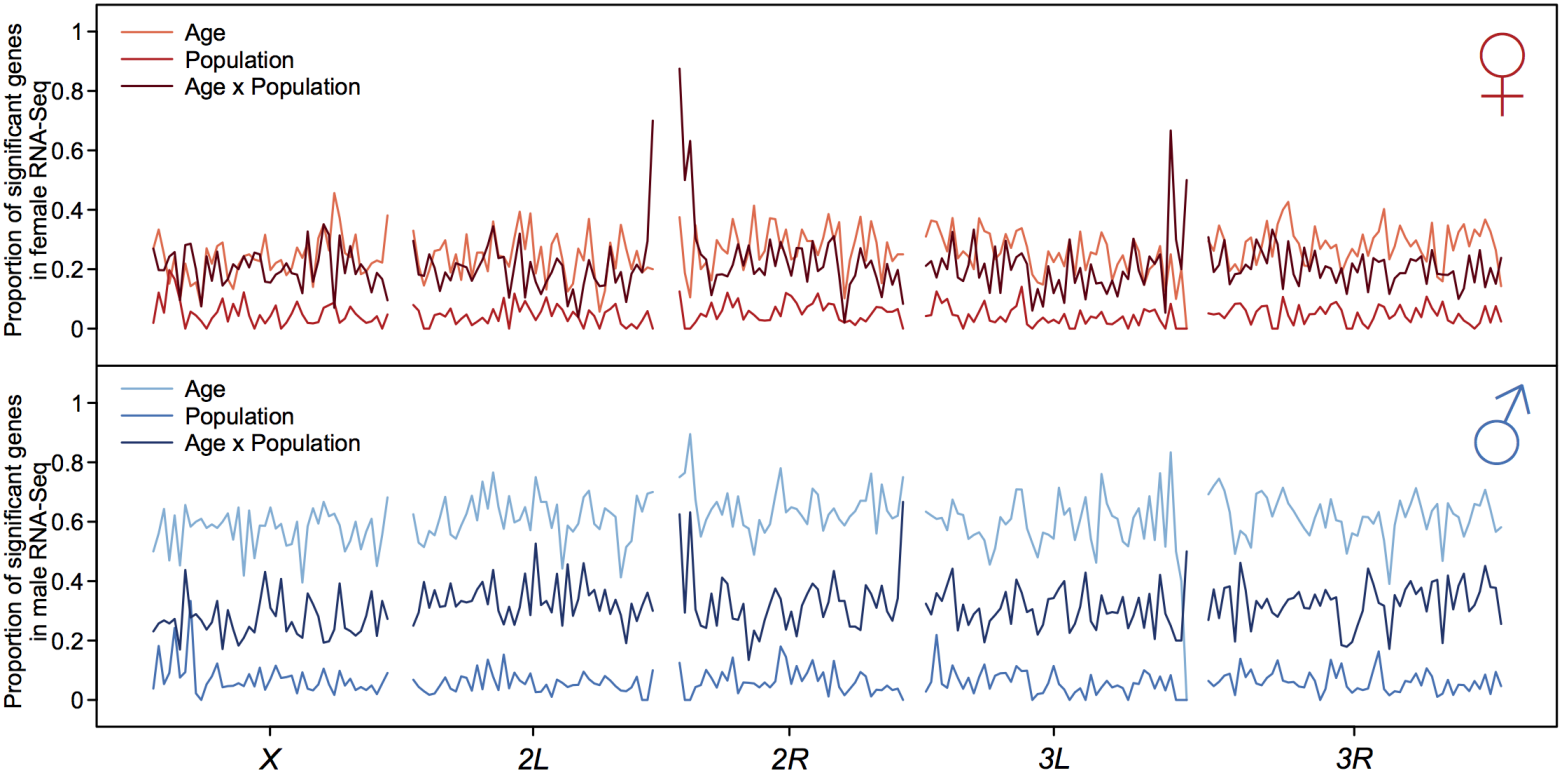
Genome-wide distribution of genes showing significant age, population, and age x population interaction effects on gene expression. The proportions of genes showing significant age, population or age x population interaction effects within 0.5Mb non-overlapping windows are plotted along the chromosome arms. Genes are located by their mid-gene coordinates. The top and bottom panels are the distributions for females and males, respectively.

We first examined the genomic distribution of genes with significant main effects of age, population, and the age by population interaction (Fig 3). We plotted the fraction of significant genes in each category in non-overlapping 0.5 Mb windows, and found they were largely uniformly distributed across the genome, with a few notable exceptions. In females, there is strong enrichment of genes significant for the age by population interaction near the centromeres of chromosomes *2L* and *2R* and also near the centromere of chromosome *3L*. In males, the enrichment is near the centromere and telomere of chromosome *2R*. The genes in these regions do not have anything obvious in common, nor are they members of gene families (S7 Table).

Genes whose expression changes with age are candidate genes affecting lifespan as well as biomarkers of aging. A large fraction of the genome– 27% in females and 61% in males—changes expression with age (Table 1), consistent with previous studies [23,130,134–136]. We performed GO enrichment analyses for the genes that were up- and down-regulated with age, separately for females (S8 Table) and males (S9 Table). In females, genes up-regulated with age were highly enriched for GO terms associated with immune response, stress response, defense response and detoxification of xenobiotics, while genes that were down-regulated with age were highly enriched for GO terms associated with mitochondrial function and oxidative phosphorylation. Genes that were up- and down-regulated with age in males were enriched for the same GO categories as for females, as expected since 74% of the genes that changed with age in females and males were the same (Table 1, S9 Table). In addition, genes that were up-regulated with age in males were highly enriched for GO categories associated with morphogenesis and development, including development of the nervous system; regulation of metabolism, gene expression and protein synthesis; signal transduction, mitosis, DNA repair, and programmed cell death. In addition, 41 up-regulated genes were significantly enriched for the GO terms “determination of adult life span”, “aging” and “multicellular organismal aging” (S9 Table). Genes that were down-regulated with age in males were also enriched for GO terms associated with metabolism and catabolism (S9 Table).

Genes with significant changes in expression between the B and O populations are candidate QTLs affecting variation in lifespan. In contrast to the large fraction of the genome associated with expression changes with age, only ~5% of the genome fell into this category, and these genes were largely different in females and males (Table 1). In females, genes down-regulated in B lines were enriched for GO terms associated with gene expression, protein synthesis, mitosis, metabolism, RNA binding and mitochondrial function; while genes up-regulated in B lines were enriched for GO terms associated with immune, defense and stress responses and detoxification of xenobiotics (S8 Table). In males, genes down-regulated in B lines were enriched for GO terms associated with detoxification of xenobiotics while genes up-regulated in B lines were enriched for GO terms associated with morphogenesis and development (S9 Table).

Transcripts exhibiting the signature of postponed senescence are those for which there is a change in gene expression of B lines with age but this change is attenuated but in the same direction in the O lines, and further the gene expression of older O lines remains similar to that of young B lines. Genes exhibiting this signature of postponed senescence will be among those with a significant age by population interaction term. The population by age interaction term was significant for 3,171 genes in females and 4,998 genes in males (Table 1). In females, genes with significant age by population interactions were enriched for GO terms involving the plasma membrane and development and function of the nervous system (S8 Table). In males, genes with significant age by population interactions were enriched for GO terms associated with mitochondrial function, oxidation reduction and amine metabolism (S9 Table).

Finally, we extended the analysis to previously unannotated genes, of which there were 1,950 in females and 2,299 in males (S10 and S11 Tables). Many of the novel genes were located in heterochromatic regions: 1,104 (57%) in females and 1,311 (57%) in males. Very few of the female novel genes were significantly associated with age (100, 5.1%), population (0) and the age by population interaction (29, 1.4%) (S10 Table). In contrast, many of the male novel genes were significantly associated with age-specific gene expression changes (1,037, 45.1%), although very few were associated with population (15, 0.65%) and the age by population interaction (50, 2.17%) (S11 Table).

### Candidate genes for postponed senescence

Causal variants affecting postponed reproductive senescence, increased lifespan and other traits that are among the list of variants that are divergent in allele frequency between the B and O populations. However, these variants often occur in local LD blocks leading to poor resolution of individual genes—particularly within the 2.6 Mb region at the tip of the *X* chromosome. Further, the genes with these divergent variants may be associated with other traits that have evolved as a correlated response to selection for postponed selection in the O lines and are not necessarily associated with differences in lifespan between the B and O lines. Candidate genes affecting increased lifespan and postponed senescence are also among the transcripts with significant age by population interactions. However, gene expression analyses alone cannot distinguish between expression changes causing phenotypic divergence in lifespan from those that are a consequence of phenotypic divergence, and a *cis*-regulatory change in expression of gene can cause *trans*-regulatory changes in gene expression of other genes, leading to correlated gene expression modules [132,137,138]. Therefore, we hypothesized that integrating results from sequence divergence gene expression divergence would enable us to identify the top candidate genes affecting postponed senescence and increased lifespan as those significant in both analyses.

**Fig 4:**
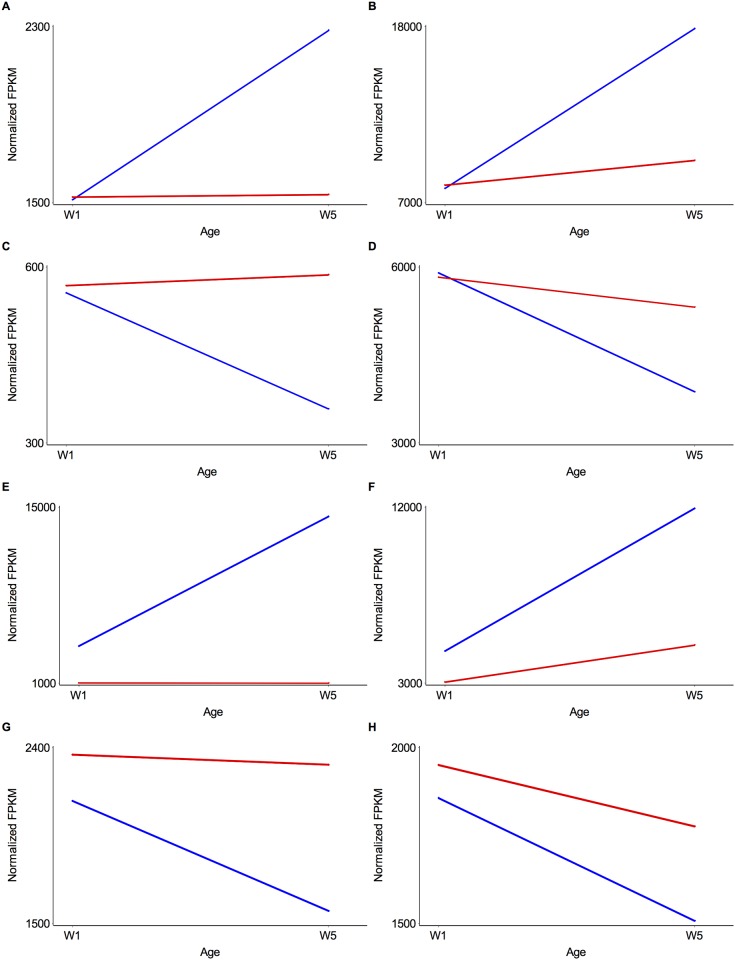
Interaction plots showing signatures of postponed senescence. Each plot shows the mean gene expression levels in normalized fragments per kilobase of exon per million fragments mapped (FPKM) at week 1 (W1) and week 5 (W5) in the B population (blue) and the O population (red). All examples are for male gene expression. See text for the description of the groups. (**A**) Group 1: *CG6188* (interaction FDR = 5.57E-04). (**B**) Group 2: *CG2233* (interaction FDR = 9.89E-06). (**C**) Group 3: *Sfp53D* (interaction FDR = 4.83E-06). (**D**) Group 4: *CG31198* (interaction FDR = 8.72E-05). (**E**) Group 5: *CR45054* (interaction FDR = 5.01E-05). (**F**) Group 6: CG6910 (interaction FDR = 3.63E-06). (**G**) Group 7: *CG2930* (interaction FDR = 2.10E-05). (**H**) Group 8: *Vap-33B* (interaction FDR = 4.22E-03).

Not all significant age by population interactions in the gene expression analyses are consistent with the pattern of gene expression difference between the O and B lines expected from postponed senescence. We therefore filtered these genes by requiring that they fall into one of eight interaction groups consistent with postponed senescence (Fig 4). We defined interaction groups 1 and 2 as those for which there was no significant difference in expression between the B and O populations at week 1, expression in the B populations significantly increases in week 5, and expression in the O populations either remains constant (group 1) or changes marginally at week 5 (group 2). Similarly, we defined interaction groups 3 and 4 as those for which there is again no significant difference in expression between the B and O populations at week 1, expression in the B populations is significantly decreased in week 5, and expression in the O populations either remains constant (group 3) or changes marginally at W5 (group 4). Interaction groups 5 and 6 are those for which there is a significant difference in expression between the B and O populations at both weeks, but the difference is larger at week 5 than week 1, expression in the B lines is significantly increased at week 5, and expression in the O lines either remains constant (group 5) or changes marginally at week 5 (group 6). Finally, interaction groups 7 and 8 are those for which there is a significant difference in expression between the B and O populations at both weeks, but this difference is larger at week 5 than week 1, expression in the B populations is significantly decreased at week 5, and expression in the O populations either remains constant (group 7) or changes marginally at W5 (group 8). Applying these filters reduced the number of candidate genes to 687 in females (S12 Table) and 1,459 in males (S13 Table). The vast majority of these genes were in interaction groups 1–4 (94.5% in females and 94.3% in males).

We next required that a variant from the divergence analysis occurred within 1 kb of the genes with expression changes consistent with postponed senescence and increased lifespan. A total of 98 genes in females and 175 genes in males remained after applying this filter (S14 and S15 Tables). These genes were not enriched for any GO terms. Rather, they spanned the diversity of biological functions previously associated with aging, including the regulation of metabolism and macromolecule biosynthesis, immune/defense response, stress resistance, reproduction, mitochondrial function, oxidation-reduction, DNA repair and replication, and regulation of gene expression. Many of these genes affect developmental processes, including development and function of the nervous system, none of which have previously been associated with lifespan. Possibly the same genes that act early in development to build organismal structures are also responsible for the long-term maintenance of these structures in adults. Approximately one-third of the candidate genes associated with postponed senescence have no known biological functions. Finally, only 20 of these genes are in common between males and females.

## Discussion

We have characterized the phenotypic, genetic and genomic divergence of lines subjected to long-term laboratory evolution for postponed reproductive senescence [109]. The O lines live nearly twice as long as the B lines, have increased productivity at all ages, and exhibit postponed senescence for other phenotypes not under direct selection. We determined the genomic DNA sequences of the O and B lines to assess the pattern and magnitude of genetic differentiation between them. Of the ~18,000 genes in the *Drosophila* genome, ~11% have a variant that is genetically divergent between the O and B populations at our reporting threshold of *P* < 10^−3^ (FDR = 0.068), many of which are in a 2.6 Mb region of the X chromosome that appears to have undergone a hard selective sweep [128].

We performed RNA sequencing of the O and B lines at one and at five weeks of age, and used ANOVA to partition the variation in gene expression for each expressed transcript into the main effects of age and population, and the age by population interaction, separately for each sex. Consistent with previous studies [23,130,134–136], we found that a substantial fraction of the genome exhibits significant (FDR < 0.05) changes in gene expression with age. Intriguingly, transcriptional divergence between the O and B populations was modest (5–6% of the genome) with respect to the main effect of population, but much greater (20–31%) for the age by population interaction. This suggests that transcriptional divergence between the O and B populations is age-specific, as would be expected for transcripts with less change with age in the O lines relative to the B lines (*i*.*e*., postponed senescence). We identified the subset of genes with significant age by population interactions with gene expression signatures consistent with postponed senescence, and the subset of those genes that are candidates for postponed senescence because they are also genetically divergent between the B and O population– 98 genes for females and 175 genes for males.

Several of these candidate genes have been previously associated with *Drosophila* lifespan. We identified *CG10383*, which encodes a hydrolase, as a candidate gene for postponed senescence and increased lifespan in both sexes. Over-expression of *CG10383* in the nervous systems extends lifespan [139]. *Catalase* (*Cat*) and *Autophagy-related 7* (*Atg7*) were among the candidate genes affecting lifespan in females. Null mutations of *Cat* [65] and *Atg7* [140] reduce lifespan. Candidate genes in males included *methuselah-like 8* (*mthl8*), *molting defective* (*mld*, also known as *DTS-3*), *CTP*:*phosphocholine cytidylyltransferase 1* (*Cct1*), *Peptidoglycan recognition protein LF* (*PGRP-LF*) and *brummer* (*bmm*). *mthl8* is annotated to affect lifespan by virtue of its homology with *mth* (*methuselah*), which encodes a G-protein coupled receptor that, when down-regulated, increases lifespan [114]. *mld* (*DTS-3*) encodes a protein with Krüppel Zn-finger domains and is involved in ecdysone biosynthesis. A dominant temperature sensitive allele of *mld* has been associated with a female-specific increase in lifespan [141]. The observation that *mld* is a male-specific candidate gene in this study is not surprising, since we have previously observed that the same mutation affecting lifespan can have different sex-specific effects depending on the genetic background, and that different mutations in the same gene and genetic background can have variable and sometime opposite effects on longevity [131,142]. *Cct1* [87] and *PGRP-LF* [139] were both identified in large scale screens for genes with increased lifespan. *bmm*, which encodes a triglyceride lipase, was found to be up-regulated on starvation in a genome wide transcriptome analysis comparing fed and food-deprived flies [143]. Subsequent analyses showed that a mutation in *bmm* had a context-dependent effect on lifespan, with decreased lifespan under fed conditions, but increased lifespan when flies were starved [143].

Several of the candidate genes for postponed reproductive senescence and increased lifespan were associated with a variant from the divergence analysis with a *P*-value exceeding a Bonferroni correction for multiple tests: *CG18031*, *CG42340*, *CG11378*, *CG3699* and *mus81* in females; *Insulin-like peptide 6* (*Ilp6*), *Cytochrome P450-4d1* (*Cyp4d1*), *silver* (*svr*), *female sterile (1) Nasrat* (*fs(1)N*), *CG2854*, *CG13868*, *CG6428*, *CG7713*, *Centaurin gamma 1A* (*CenG1A*) and *CG33307* in males. This study represents the first biological functional annotation for the majority of these genes (*CG18031*, *CG42340*, *CG11378*, *CG3699*, *Cyp4d1*, *CG2854*, *CG13868*, *CG6428*, *CG7713*, *CG33307*). While the others have not been implicated to affect lifespan previously, most are plausible candidates because they perform similar roles as other genes known to affect aging. *mus81* plays a role in DNA repair [144]. *Ilp6* is thought to be an insulin receptor and affects adult body size by regulating post-feeding growth [145] as well as growth under conditions of nutritional deprivation [146]. *svr* encodes Carboxypeptidase D [147] and has pleiotropic effects on imaginal disc-derived wing morphogenesis, long term memory, stress response and phagocytosis [148,149]. *CenG1A* is a member of the gamma subgroup of the Centaurin superfamily of small GTPases and is a Phosphoinositide-3-kinase enhancer (PIKE) protein thought to regulate ecdysone signaling-dependent second to third instar larval transition [150]. *fs(1)N* is an unexpected candidate affecting male postponed senescence since it in involved in oogenesis and highly expressed in female reproductive tissues [121]. However, it does have a low level of expression in testes [121].

The genetic architecture of *Drosophila* lifespan is highly sex-specific [24–27,29,130,131,142], as indeed is the majority of the candidate genes identified in this study. However, 20 of our top candidate genes (including the above-mentioned *CG10383*) were found in both sexes. Again, this study represents the first biological functional annotation for most of these genes (*CG12253*, *CG5991*, *CG2233*, *CG3604*, *Organic anion transporting polypeptide 30B* (*Oatp30B*), *CG6357*, *CG11395*, *CG30098*, *CG32061*, *CG42557*, *CG43175*, *CR45054*, *CR45272*); and the other genes common to males and females are plausible candidates. *Cecropin A2* (*CecA2*) is involved in antibacterial humoral response [151–153], as is *Ras-like protein A* (*RalA*) [154]. *RalA* also regulates polar-cell differentiation during oogenesis [155]. *fat* (*ft*) is involved in cadherin and calcium ion binding and acts to regulate planar cell polarity in the wing [156] and body hair [157]. *ft* is also a tumor suppressor and as such acts to regulate growth [158]. *multiple wing hairs* (*mwh*) encodes a G protein binding domain-formin homology 3 (GBD-FH3) domain protein that acts downstream of the planar cell polarity pathway to regulate wing hair development [159,160]. *exit protein of rhodopsin and TRP* (*Xport*) is a chaperone for the transient receptor potential (TRP) channel and its G-protein coupled receptor, rhodopsin (Rh1) and interacts with both Trp and Rh as well as the small heat shock proteins Hsp27 and Hsp90 [161]. *shrub* (*shrb*, also known as *ESCRT*) has pleiotropic effects on multiple biological process, including autophagy [162], nervous system development [163–166], negative regulation of mycobacterial growth [167] and growth of the female germ line [168,169].

The candidate genes affecting postponed senescence and increased lifespan identified in this study are a rich resource for future functional validation. Many of these genes have human orthologs and may advance our understanding of ‘public’ mechanisms of aging [3,43]. Although we used the lowest *P*-value from the genetic divergence analysis as a filter to integrate with the gene expression data, these polymorphisms are not necessarily the causal ones. However, many are indeed in transcriptional start and end sites of the genes exhibiting transcriptional signatures of postponed senescence, a hallmark of *Drosophila cis*-eQTLs [170]. Determining which polymorphisms are causal will be a critical step towards functional analyses of their pleiotropic effects on fitness and insights about why they remain segregating in nature, providing specific examples of general evolutionary explanations.

## Supporting Information

S1 Table Mixed model analyses of variance of lifespan and senescence for feeding behavior in the CAFÉ assay, phototaxis, chill coma recovery time and productivity in the B and O lines. Population and Age are fixed effects, the rest are random. df: degrees of freedom; MS: Type III mean squares, F: F ratio statistic; *P*: **P**-value.
(DOCX)

S2 Table Divergence in allele frequency between O and B lines. All frequencies are estimated with respect to the allele that is more frequent in the O lines, which is indicated by the column "Allele for frequency estimation".
(XLSX)

S3 Table Biological Process Gene Ontology (GO) enrichment analysis of genes divergent in O and B lines (excluding the first 2.6Mb of the *X* chromosome). An enrichment score of 5 (corresponding to a geometric mean normalized P-value threshold of 1E-5) was used as the significance cutoff from the functional annotation cluster analysis.
(XLSX)

S4 Table Gene expression data for all samples. B1-B5 are the five B lines and O1- O5 the five O lines. W1 and W5 denotes weeks one and five, respectively. F indicates females and M males, 1 and 2 are the two biological replicates per population, week and sex. Tab A gives raw expression data in FPKM and Tab B the geometric mean normalized expression counts.
(XLSX)

S5 Table *P*-values and FDR from ANOVA of female gene expression and summary statistics. The variant with the smallest *P*-value from the genetic divergence analyses is also given.
(XLSX)

S6 Table *P*-values and FDR from ANOVA of male gene expression and summary statistics. The variant with the smallest *P*-value from the genetic divergence analyses is also given.
(XLSX)

S7 Table Gene in regions enriched for transcripts with significant Age and/or Population × Age terms. (XLSX)

S8 Table Gene Ontology (GO) enrichment analyses of female gene expression data. An enrichment score of 5 (corresponding to a geometric mean normalized P-value threshold of 1E-5) was used as the significance cutoff from the functional annotation cluster analysis. (A) Age analyses. (B) Population analyses. (C) Population and age and population× age analyses.
(XLSX)

S9 Table Gene Ontology (GO) enrichment analyses of male gene expression data. An enrichment score of 5 (corresponding to a geometric mean normalized P-value threshold of 1E-5) was used as the significance cutoff from the functional annotation cluster analysis. (A) Age analyses. (B) Population analyses. (C) Population and age and population× age analyses.
(XLSX)

S10 Table *P*-values and FDR from ANOVA of female gene expression of novel genes and summary statistics. W1: Week 1; W5: Week 5.
(XLSX)

S11 Table *P*-values and FDR from ANOVA of female gene expression of novel genes and summary statistics. W1: Week 1; W5: Week 5.
(XLSX)

S12 Table Transcripts exhibiting signatures of postponed senescence in females. (XLSX)

S13 Table Transcripts exhibiting signatures of postponed senescence in males. (XLSX)

S14 Table Candidate genes for postponed senescence and increased lifespan in females. (XLSX)

S15 Table Candidate genes for postponed senescence and increased lifespan in males. (XLSX)
