# Supplementary material for: The Genomic Basis of Postponed Senescence in Drosophila melanogaster
Source: PLoS One. 2015 Sep 17;10(9):e0138569. doi: 10.1371/journal.pone.0138569 (PMC4574564; doi:10.1371/journal.pone.0138569)
Supplement: S1 Table — Population and Age are fixed effects, the rest are random. df: degrees of freedom; MS: Type III mean squares, F: F ratio statistic; P: P-value. (DOCX) [file pone.0138569.s001.docx]

**S1 Table.** Mixed model analyses of variance of lifespan and senescence for feeding behavior in the CAFÉ assay, phototaxis, chill coma recovery time and productivity in the B and O lines. Population and Age are fixed effects, the rest are random. df: degrees of freedom; MS: Type III mean squares, F: F ratio statistic; *P*: *P*-value.

| **Trait** | **Sex** | **Source** | **df** | **MS** | **F** | ***P*** |
| --- | --- | --- | --- | --- | --- | --- |
| Lifespan | F | Population | 1 | 240561.0 | 36.59 | 3.00E-04 |
|  |  | Line(Population) | 8 | 6585.4 | 17.62 | 3.03E-23 |
|  |  | Rep(Population×Line) | 487 | 375.4 | 1.26 | 1.40E-03 |
|  |  | Error | 918 | 296.9 |  |  |
|  | M | Population | 1 | 222435.0 | 118.63 | 4.45E-06 |
|  |  | Line(Population) | 8 | 1879.0 | 6.07 | 1.79E-07 |
|  |  | Rep(Population×Line) | 485 | 310.5 | 1.19 | 1.53E-02 |
|  |  | Error | 905 | 261.9 |  |  |
| CAFÉ | F | Age | 3 | 10.665 | 28.42 | 3.82E-08 |
|  |  | Population | 1 | 4.475 | 7.77 | 2.34E-02 |
|  |  | Age×Population | 3 | 2.997 | 7.98 | 7.00E-04 |
|  |  | Line(Population) | 8 | 0.577 | 1.54 | 1.96E-01 |
|  |  | Age×Line(Population) | 24 | 0.376 | 1.08 | 3.72E-01 |
|  |  | Error | 193 | 0.348 |  |  |
|  | M | Age | 3 | 5.618 | 11.58 | 6.35E-05 |
|  |  | Population | 1 | 16.310 | 7.08 | 2.87E-02 |
|  |  | Age*Population | 3 | 8.209 | 16.93 | 3.58E-06 |
|  |  | Line(Population) | 8 | 2.332 | 4.79 | 1.30E-03 |
|  |  | Age×Line(Population) | 24 | 0.487 | 1.41 | 1.08E-01 |
|  |  | Error | 190 | 0.346 |  |  |
| Phototaxis | F | Age | 3 | 399.50 | 8.13 | 7.00E-04 |
|  |  | Population | 1 | 9067.68 | 121.14 | 4.11E-06 |
|  |  | Age×Population | 3 | 62.41 | 1.27 | 3.07E-01 |
|  |  | Line(Population) | 8 | 75.36 | 1.53 | 1.99E-01 |
|  |  | Age×Line(Population) | 24 | 49.73 | 7.3 | 6.69E-12 |
|  |  | Rep(Age×Population×Line) | 79 | 6.87 | 3.68 | 4.74E-25 |
|  |  | Error | 5,409 | 1.87 |  |  |
|  | M | Age | 3 | 416.17 | 9.45 | 3.00E-04 |
|  |  | Population | 1 | 6588.88 | 49.82 | 1.00E-04 |
|  |  | Age×Population | 3 | 91.55 | 2.08 | 1.30E-01 |
|  |  | Line(Population) | 8 | 132.86 | 3.02 | 1.71E-02 |
|  |  | Age×Line(Population) | 24 | 44.47 | 4.99 | 2.56E-08 |
|  |  | Rep(Age×Population×Line) | 80 | 8.97 | 5.15 | 5.71E-44 |
|  |  | Error | 5,286 | 1.74 |  |  |
| Chill Coma | F | Age | 3 | 2729.4 | 23.55 | 2.49E-07 |
|  |  | Population | 1 | 6507.1 | 5.47 | 4.75E-02 |
|  |  | Age×Population | 3 | 1456.1 | 12.56 | 3.87E-05 |
|  |  | Line(Population) | 8 | 1189.5 | 10.26 | 4.05E-06 |
|  |  | Age×Line(Population) | 24 | 116.0 | 4.89 | 8.82E-14 |
|  |  | Error | 1,965 | 23.7 |  |  |
|  | **Sex** | **Source** | **df** | **MS** | **F** | ***P*** |
|  | M | Age | 3 | 809.6 | 6.28 | 2.70E-03 |
|  |  | Population | 1 | 396.9 | 0.8 | 3.97E-01 |
|  |  | Age×Population | 3 | 545.4 | 4.23 | 1.55E-02 |
|  |  | Line(Population) | 8 | 494.9 | 3.84 | 4.90E-03 |
|  |  | Age×Line(Population) | 24 | 129.1 | 8.75 | 8.36E-30 |
|  |  | Error | 1,941 | 14.8 |  |  |
| Productivity | N/A | Age | 3 | 5485.2 | 29.61 | 3.10E-08 |
|  |  | Population | 1 | 46760.0 | 208.79 | 5.15E-07 |
|  |  | Age×Population | 3 | 2943.3 | 15.89 | 6.67E-06 |
|  |  | Line(Population) | 8 | 224.0 | 1.21 | 3.35E-01 |
|  |  | Age×Line(Population) | 24 | 185.3 | 2.44 | 2.00E-04 |
|  |  | Error | 360 | 75.8 |  |  |
